# The effect of premature mortality due to despair on life expectancy in Brazil

**DOI:** 10.1590/1980-549720250039

**Published:** 2025-07-21

**Authors:** Raphael Mendonça Guimarães, José Henrique Costa Monteiro-da-Silva

**Affiliations:** I Fundação Oswaldo Cruz, National School of Public Health - Rio de Janeiro (RJ), Brazil.; II University of Pennsylvania, Population Studies Center - Philadelphia (PA), United States of America.

**Keywords:** Life expectancy, Mental disorders, Opioid epidemic, Economic recession, Mortality, premature, Expectativa de vida, Transtornos mentais, Epidemia de opioides, Recessão econômica, Mortalidade prematura

## Abstract

**Objective:**

The aim of this study was to estimate the impact of deaths of despair (DoD) on life expectancy at birth and by sex in Brazil in 2019, as well as the contribution of different age groups to this loss.

**Methods:**

We used life tables from the Brazilian Institute of Geography and Statistics and cause-specific mortality data by age and sex from the Mortality Information System. A cause-deleted life table methodology was applied, assuming independence between DoD and other causes of death. The difference in life expectancy with and without DoD was decomposed by age using Arriaga’s method. DoD included deaths from suicide, intentional or accidental poisoning, and mental and behavioral disorders due to substance use.

**Results:**

In 2019, there were 23,391 DoD in Brazil (1.73% of all deaths), 89% of which were due to suicide. Removing these deaths would increase life expectancy by 0.43 years for men and 0.12 years for women, with men experiencing a 3.5 times greater impact. The 35-49 age group had the highest relative contribution, especially among men, where DoD accounted for up to 9.7% of the loss in life expectancy. The impact was more concentrated and earlier in men and more diffuse among women.

**Conclusion:**

Although lower in absolute numbers, DoD have a measurable impact on life expectancy, especially among young men. These findings highlight the need for public policies focused on suicide prevention and addressing the social determinants that sustain despair.

## RESUMO

**Objetivo:**

Estimar o impacto das mortes por desespero (DoD) na expectativa de vida ao nascer e por sexo no Brasil em 2019, bem como a contribuição das diferentes faixas etárias para essa perda.

**Métodos:**

Foram utilizados tábuas de vida do Instituto Brasileiro de Geografia e Estatística e dados de mortalidade por causa, idade e sexo do Sistema de Informação sobre Mortalidade. Aplicou-se a metodologia de tábuas de vida com causas deletadas (*cause-deleted life table*), assumindo-se independência entre DoD e outras causas. A diferença na expectativa de vida com e sem DoD foi decomposta por idade com base no método de Arriaga. Consideraram-se como DoD os óbitos por suicídio, intoxicações intencionais ou acidentais e transtornos mentais e comportamentais relacionados ao uso de substâncias.

**Resultados:**

Em 2019, ocorreram 23.391 mortes por desespero no Brasil (1,73% dos óbitos), sendo 57,8% por suicídio. A retirada dessas mortes das tábuas aumentaria a expectativa de vida em 0,43 anos para homens e 0,12 anos para mulheres — o impacto seria 3,5 vezes maior entre os homens. O grupo etário de 35-49 anos apresentou maior contribuição relativa, com destaque para homens, com essas mortes representando até 9,7% do impacto na expectativa de vida. O impacto é mais concentrado e precoce nos homens e mais difuso nas mulheres.

**Conclusão:**

As mortes por desespero, embora em menor número absoluto, têm efeito mensurável na expectativa de vida, sobretudo entre homens jovens. Esses resultados reforçam a importância de políticas públicas voltadas à prevenção de suicídio e ao enfrentamento dos determinantes sociais que sustentam o desespero.

## INTRODUCTION

The epidemiological transition has shifted the burden of disease to non-communicable conditions, increasing life expectancy globally^1^. The theoretical models of epidemiological transition, as proposed by Omran^2^, describe that in the more advanced stages, mortality continues to decline, but at a slow and gradual pace. This occurs due to the predominance of non-communicable chronic diseases, whose control is more complex and requires continuous prevention and treatment strategies. Additionally, population aging contributes to the maintenance of relatively high mortality rates. The stagnation in the decline of mortality reflects both biological and social limits to health interventions^2^. However, some countries, like the United States, have seen stagnation or decline in life expectancy despite reduced chronic disease mortality^3^. Recently, this decline has linked to premature deaths from so-called “Deaths of Despair” (DoD).

The term *deaths of despair* was introduced by Anne Case and Angus Deaton in 2015, when they analyzed the rising mortality among middle-aged white adults in the United States, particularly those without a college degree. This increase was attributed to three main causes: suicide, drug overdose, and alcohol-related liver diseases. The authors argued that these deaths reflect a prolonged social and economic crisis, characterized by labor market deterioration, family instability, and a loss of future prospects^4^. Although the term is still relatively new in the biomedical literature, there is growing consensus that the social determinants of health-including economic insecurity, unemployment, and lack of community cohesion-are critical underlying factors in this phenomenon.. Case and Deaton first identified this trend among middle-aged, non-Hispanic white men in the U.S. after 1998, attributing it to rising psychological distress^5^. Since then, the concept has been used to describe similar patterns in other contexts.

Recent research explores DoD-related mortality differentials and their impact on life expectancy. While the U.S. data highlights stagnation in longevity gains, the impact of DoD in peripheral economies like Brazil remains underexplored. Most studies on deaths of despair have focused on the United States and, more recently, other high-income countries such as Canada, the United Kingdom, Australia, and some Scandinavian countries. The impact of these deaths on life expectancy in Brazil has not yet been systematically studied, especially considering the country’s diverse population composition, regional patterns of inequality, and incomplete social protection systems. From the perspective of Brazil’s structural position in the global economy-beyond per capita income-it is a country reliant on commodity exports, dependent on external flows of capital and technology, and with low value-added production chains. Although the World Bank classifies Brazil as an upper-middle-income country, its unequal and exclusionary growth model, combined with institutional fragility, still places it in a peripheral structural position. This gap becomes even more important to investigate.. Hence, we conducted a preliminary analysis based on demographic methods to estimate the impact of deaths due to despair on adult life expectancy at birth in Brazil, according to sex, and to measure the contribution of each age group to this impact.

## METHODS

To estimate the impact of DoD on Brazil’s 2019 life expectancy, we used Institute of Geography and Statistics (IBGE) life tables by sex and death data by age and sex from the Mortality Information System (SIM). A cause-deleted life table (CDLT) was constructed to model life expectancy without DoD as a cause of death, assuming that DoD mortality is independent of other causes and does not affect their mortality rates. We used 2019 data from the Mortality Information System to analyze deaths of despair without the interference of the COVID-19 pandemic, which significantly impacted suicide and chemical poisoning rates. Studies indicate that during the pandemic, there was a considerable increase in these deaths, possibly due to social isolation and economic stress. For example, a study in Germany revealed changes in suicide rates during lockdown periods in 2020^6^. Another study in Italy reported an increase in suicide mortality in 2021, especially among men^7^. Thus, the 2019 data provide a more accurate baseline for assessing underlying trends in deaths of despair. Table 1 presents the list of DoD International Classification of Diseases, Tenth Revision codes.

**Table 1.** Causes of death and ICD-10 codes related to deaths of despair.

Group of causes	ICD-10 codes
Accidental or intentional poisoning and poisoning of undetermined intent from drug exposure; drugs in the blood	X40-X44, X60-64, Y10-Y14; R78.1-R78.5
Drug-induced illnesses	D52.1, D59.0, D59.2, D61.1, D64.2, E06.4, E16.0, E23.1, E24.2, E27.3, E66.1, G21 .1, G24.0, G25.1, G25.4, G25.6, G44.4, G62.0, G72.0, I95.2, J70.2-J70.4, K85.3, L10.5 , L27.0, L27.1, M10.2, M32.0, M80.4, M81.4, M83.5, M87.1, R50.2
Mental/behavioral disorders due to drugs	F11.0-F11.5, F11.7-F11.9, F12.0-F12.5, F12.7-F12.9, F13.0-F13.5, F13.7-F13.9, F14.0-F14.5, F14.7-F14.9, F15.0-F15.5, F15.7-F15.9, F16.0-F16.5, F16.7-F16.9, F18.0-F18.5, F18.7-F18.9, F19.0-F19.5, F19.7-F19.9
Alcohol-induced illnesses	E24.4, G31.2, G62.1, G72.1, I42.6, K29.2, K70.0-K70.4, K70.9, K73.4, K85.2, K86.0, R78.0
Mental/behavioral disorders due to alcohol	F10.0-F10.9
Accidental or intentional poisoning and poisoning of undetermined intent from alcohol exposure	X40-45, X65, Y10-15, Y45, Y47, Y49
Suicide	X66-X84, Y87.0

ICD-10: International Classification of Diseases, Tenth Revision.

Considering that the force of mortality of the scenario in which DoD is eliminated as a cause of death is proportional to the force of mortality of all causes combined, the following relation holds (Equation 1):

$$\dot{n}P_{X}^{\left(-i\right)}={\left[nP_{X}^{i}\right]}^{R^{\left(-i\right)}}={\left[nP_{X}^{i}\right]}^{\left(1-{nD}_{x}^{i}/nD_{x}\right)}$$ (1)

Where:

$\dot{n}P_{X}^{\left(-i\right)}$: the probability of surviving in the age interval x to x + n in the CDLT.

Where cause i was deleted as a cause of mortality:

$nP_{X}^{i}$: the probability of surviving in the age interval x to x + n in the overall mortality life ta

ble from IBGE;

${nD}_{x}^{i}$: the observed number of DoD for ages x to x + n;

$nD_{x}$: the observed number of deaths from all causes for ages x to x + n;

$R^{\left(-i\right)}$: the proportion of deaths from all causes different from DoD.

Then, the CDLT survival function $l_{x+n}^{\left(-i\right)}$ can be estimated as follows (Equation 2):

lx+n(-i)*n̿px(-i)*lx(-i) (2)

We estimate the CDLT average person-years lived by those who die in any age interval between ages x and x + n (${\dot{n}}^{a_{x}^{\left(-i\right)}}$) by following the graduation method proposed by Preston^8^, and from these life table functions, we can retrieve the life expectancy at age x for the CDLT ($e_{x}^{\left(-i\right)}$). Finally, we decompose the difference in life expectancy at birth to estimate the contributions of differences in age-specific mortality rates (nΔx) between the DoD CDLT ($e_{0}^{\left(-i\right)}$) and the IBGE life table ($e_{0}$) for men and women using Arriaga’s decomposition method^9^. Following this method, we have Equation 3:

$$e_{0}^{\left(-i\right)}-e_{0}=\sum_{x}n\Delta x$$ (3)

Where (Equation 4):

$$n\Delta x=\frac{l_{x}}{l_{0}}*\left(\frac{nL_{x}^{\left(-i\right)}}{l_{x}^{\left(-i\right)}}-\frac{nL_{x}}{l_{x}}\right)+\frac{T_{x+n}^{\left(-i\right)}}{l_{o}}*\left(\frac{l_{x}}{l_{x}^{\left(-i\right)}}-\frac{l_{x+n}}{l_{x+n}^{\left(-i\right)}}\right)$$ (4)

In which:

l_x_ and nL_x_: respectively, is the number of individuals at age x in the life table synthetic cohort and the person-years lived between ages x and x + n from the IBGE life table;

$l_{x}^{\left(-i\right)}$ and $nL_{x}^{\left(-i\right)}$: their equivalents from the CDLT;

$T_{x+n}^{\left(-i\right)}$: the number of person-years lived from age x + n and above in the CDLT.

The study adhered to the Declaration of Helsinki’s ethical standards. Using publicly available, non-identified mortality data required no institutional review board approval or informed consent, ensuring compliance with ethical and legal procedures.

### Data availability

Data availability statement: The entire dataset supporting the results of this study was published in the article itself.

## RESULTS

In 2019, Brazil recorded 23,391 DoD, accounting for 1.73% of total deaths. The 2019 Synthetic Life Table for Brazil highlights significant sex-based disparities in how deaths of despair (DoD) affect life expectancy. Among men, DoD accounted for 2.53% of total mortality, compared to 0.75% among women. Suicides represented the majority of these deaths, comprising 57.8% (n=13,520). On average, DoD shortened male life expectancy by 0.43 years (0.64%) and female life expectancy by 0.12 years (0.15%), meaning men faced an impact 3.54 times greater than women (Table 2). While men experienced a higher absolute reduction in years, the proportional impact also stands out. In several male age groups, removing deaths of despair led to a relative increase of over 9% in life expectancy at that age-underscoring the preventable nature of these deaths and their critical influence on male longevity.

**Table 2.** Life tables with deleted cause and decomposition by age group and sex. Brazil, 2019.

Sex	x	_n_ **D**_x_ **[all causes]**	_n_ **D**_x_ **[DoD]**	R_i_	l_x_	e_x_	l_x_ [DoD Deleted]	e_x_ [DoD Deleted]	Delta (e_x_[DoD Deleted] - e_x_)	
									n	%
Male	0	19,437	0	0.000	100,000	73.06	100,000	73.49	0.000	0.00
	1	3,275	2	0.001	98,715	73.01	98,715	73.44	0.000	0.02
	5	1,775	2	0.001	98,491	69.17	98,491	69.61	0.000	0.02
	10	2,519	89	0.035	98,358	64.26	98,358	64.70	0.004	1.00
	15	13,318	832	0.063	98,186	59.37	98,192	59.80	0.026	6.04
	20	20,335	1,319	0.065	97,452	54.80	97,504	55.21	0.038	8.89
	25	18,620	1,327	0.071	96,343	50.40	96,466	50.77	0.038	8.91
	30	19,702	1,486	0.075	95,241	45.95	95,441	46.29	0.039	9.04
	35	22,644	1,747	0.077	94,072	41.49	94,357	41.79	0.041	9.63
	40	25,925	1,782	0.069	92,718	37.06	93,103	37.32	0.042	9.67
	45	31,381	1,800	0.057	91,000	32.71	91,496	32.93	0.042	9.72
	50	42,668	1,902	0.045	88,642	28.51	89,259	28.69	0.039	9.15
	55	54,522	1,870	0.034	85,349	24.51	86,089	24.65	0.035	8.26
	60	66,424	1,465	0.022	80,850	20.73	81,702	20.83	0.026	5.97
	65	74,116	1,226	0.017	74,859	17.18	75,776	17.25	0.021	4.87
	70	76,165	821	0.011	66,842	13.92	67,788	13.98	0.014	3.33
	75	76,965	528	0.007	56,171	11.07	57,073	11.11	0.009	2.12
	80	173,884	670	0.004	42,977	8.68	43,747	8.72	0.014	3.36
							Total Difference		0.43	100%
Female	0	15,658	0	0.000	10,0000	80.09	100,000	80.21	0.000	0.00
	1	2,547	1	0.000	98,902	79.98	98,902	80.10	0.000	0.05
	5	1,390	1	0.001	98,724	76.12	98,724	76.24	0.000	0.04
	10	1,704	107	0.063	98,626	71.19	98,626	71.31	0.005	3.78
	15	3,381	308	0.091	98,517	66.27	98,524	66.39	0.012	9.63
	20	4,622	373	0.081	98,315	61.40	98,341	61.50	0.012	9.66
	25	5,281	341	0.065	98,070	56.54	98,115	56.64	0.011	8.77
	30	7,243	333	0.046	97,766	51.71	97,831	51.80	0.009	7.78
	35	10,385	404	0.039	97,352	46.92	97,435	47.00	0.010	8.24
	40	13,760	384	0.028	96,779	42.18	96,884	42.25	0.010	7.85
	45	17,882	430	0.024	95,926	37.53	96,054	37.59	0.011	9.26
	50	24,751	390	0.016	94,612	33.02	94,769	33.06	0.009	7.81
	55	33,486	349	0.010	92,706	28.64	92,890	28.68	0.008	6.48
	60	43,422	302	0.007	89,984	24.43	90,190	24.46	0.007	5.38
	65	52,849	238	0.005	86,062	20.42	86,287	20.44	0.005	4.30
	70	59,744	175	0.003	80,278	16.70	80,512	16.72	0.004	3.22
	75	69,156	139	0.002	71,828	13.36	72,062	13.37	0.003	2.32
	80	236,229	248	0.001	60,067	10.46	60,283	10.47	0.007	5.43
							Total Difference		0.12	100%

_n_D_x_: Number of deaths between age x and x+n; l_x:_ Number of survivors at age x; e_x_: Life expectancy at age x; R_i_: (Ratio DoD/All causes).

Gains in life expectancy from the exclusion of deaths of despair are nearly negligible during early childhood (ages 0-5) and remain modest after age 70 for both men and women. This pattern reinforces the low incidence of such deaths at the extremes of the life course. In contrast, most of the impact on life expectancy concentrates between ages 20 and 50, particularly among men. This suggests that deaths of despair disproportionately affect individuals during the most productive years of life, leading not only to a significant loss of potential years of life but also to substantial social and economic consequences. Among men, the largest gains appear between ages 25 and 50, especially from 30 to 45, where both absolute and relative deltas peak. Among women, although the highest relative contribution occurs between ages 15 and 20, the values remain considerably lower and more scattered across age groups.

The ratio between deaths of despair and total deaths further underscores this sex-based difference. Among men, the highest values consistently cluster between ages 30 and 45, with peaks at 35 and 40 years. Among women, the ratio reaches its maximum at age 15 but declines rapidly in subsequent years. In other words, while the impact of deaths of despair among men is concentrated within specific age ranges (25-50 years), among women it is more evenly distributed throughout the reproductive and adult life span, with lower absolute values and a smoother curve-suggesting distinct patterns of exposure and vulnerability. These findings indicate that deaths of despair exert a more intense and prolonged influence on men’s life trajectories, particularly during young and economically productive adulthood, whereas for women the impact tends to occur earlier and with less overall magnitude.

The gender gap in life expectancy is 7.03 years at birth, narrowing to 6.72 years when excluding DoD. Younger men are disproportionately affected: 35-49-year-olds account for 6.7% of male deaths from DoD, compared to 2.9% for women. This age group contributes 28.2% of male DoD deaths and 26.9% of female DoD deaths. Among men, the 35-49 age group accounts for 29% of the life expectancy difference linked to DoD, compared to 25% for women. While older adults (80+) are less affected, DoD contributes 5.43% of deaths among women and 3.36% among men in this group. The number of survivors at age x (lx) supports this analysis, revealing marked differences between men and women in Brazil in 2019. From birth through older ages, women consistently show higher survival rates. At age 60, for instance, approximately 90,000 women remain alive compared to 81,000 men. This gap widens at more advanced ages: by age 80, about 60,000 women survive, while only 43,000 men do. These figures reflect both the greater female longevity and the higher levels of premature mortality among men-partly driven by external causes such as deaths of despair.

## DISCUSSION

The nature of the relationship between mortality and life expectancy is complex. Transformations in general mortality regimes create essential differences in the probability of death by age group. When younger groups contribute more significantly to deaths, there is an increase in early mortality. Since 2014, life expectancy in the U.S. has fallen by 3 years. Mortality increases are concentrated among Americans in “middle age,” or between 25 and 64 years^9^. The most significant relative gains in mortality typically occurred in more vulnerable populations, particularly among middle-aged men, with less education and in rural areas or other settings with evidence of economic hardship or diminished social capital^10^. This pattern highlights the role of DoD, which disproportionately affects vulnerable populations and demands detailed studies in fragile economies.

DoD patterns vary globally; in the U.S., opioid poisonings dominate, whereas suicides are the primary contributor in Brazil^11^. Our study offers a novel analysis of the DoD’s impact on life expectancy, focusing on Brazil. Although the overall impact is modest, suicides dominate this category, reshaping perspectives on their contribution to mortality. Men aged 35-49 account for 30% of life expectancy gains, emphasizing younger populations’ significant influence. We believe that the etiology of this new phenomenon has a social and economic nature. We ratify that the study of DoD needs to be carried out considering more complex analyses of premature deaths. While further research is essential, the social and economic roots of DoD are evident, requiring complex analyses that incorporate multiple causes and competing risks^12^.

We highlight that the composition of the group of DoD varies substantially by location. Some countries have a more outstanding contribution from poisonings, as is the case of the U.S. concerning the indiscriminate use of opioids. This distinction has been the subject of recent debate, and some experts propose abandoning the term^13^. Although helpful in drawing attention to social inequalities, the expression oversimplifies distinct realities. Evidence suggests that these events have specific risk factors and distinct trajectories, and are not adequately explained by a general sense of despair.. However, in Brazil, the most significant contribution to this group comes from suicides^14^.

Persistent inequalities by race and social class potentially create differentials for specific subgroups, such as their role in other outcomes^15^. In this regard, it is also worth mentioning that Brazil has some differences regarding the profile of groups most vulnerable to deaths from despair, already documented in the literature^16^ ^,^ ^17^ ^,^ ^18^. Therefore, the extrapolation of biological aspects to social and cultural contexts will enrich the clinical approach to preventive measures. Attention to social and structural factors will undoubtedly give the mental health approach a valuable contribution to the diagnosis of risk groups and early interventions that will prevent these preventable deaths and prolonged disabilities. Still, the circumstances call for caution in the more general analysis of mortality and the decline in life expectancy.

A key limitation of this study involves the completeness and accuracy of data on the underlying cause of death recorded in the Mortality Information System (SIM), particularly for external causes such as suicides and poisonings. These categories are especially prone to underreporting, misclassification, and diagnostic uncertainty-often resulting from social stigma, insufficient investigation, or difficulty in distinguishing between accidents and intentional acts. In some cases, suicides may be incorrectly coded as undetermined causes or accidents, which undermines the reliability of estimates related to deaths of despair. This issue likely varies considerably across federal units, reflecting regional disparities in the quality of health surveillance and in access to death verification systems such as the Services for Death Verification (SVOs). For this reason, we conducted the analysis at the national level without disaggregating by smaller geographic units such as states or municipalities.

Ultimately, deaths of despair reflect not only specific clinical conditions, but also a broader context of hopelessness shaped by the erosion of social networks, economic insecurity, and a loss of future prospects^19^. This framework aligns with Link and Phelan’s fundamental cause theory, proposed in 1995, which argues that social inequalities continuously generate and sustain health disparities over time, even as the specific risk mechanisms evolve^20^.

Both models emphasize the social determinants of health, highlighting how factors such as income, education, and access to resources play a decisive role in the distribution of disease and mortality. The key difference lies in their analytical scope: while the fundamental cause theory explains persistent patterns of inequality across a range of health outcomes, the deaths of despair model focuses on a specific set of mortality causes linked to the collapse of expectations for a dignified life. Together, these frameworks offer valuable insights into how social precarity translates into biological suffering and premature death.. Ultimately, the discussion on social determinants continues to be central to causality models in health. It includes strengthening social protection systems and political changes that support the State and, simultaneously, are sensitive to social issues.
